# Poor Mental Health Days and Depression by Farming Occupation and Sociodemographic Factors: BRFSS 2019 Data from 13 States

**DOI:** 10.1080/1059924X.2025.2582655

**Published:** 2025-11-06

**Authors:** Jeanne M. Ward, John R. Blosnich

**Affiliations:** a Social Work, Indiana University School of Nursing, Indianapolis, IN, USA; b Suzanne Dworak-Peck School of Social Work, University of Southern California, Los Angeles, CA, USA; c Center for Healthcare Evaluation, Research, and Promotion, VA Pittsburgh Healthcare System, Pittsburgh, PA, USA

**Keywords:** Agriculture, BRFSS, health promotion, mental health, public health, suicide prevention

## Abstract

**Background:**

Farmers are disproportionately affected by suicide, which is frequently preceded by signs of poor mental health. Findings on mental health outcomes among individuals in farming occupations are mixed. This analysis of a major national dataset sought to identify the sociodemographic factors related to poor mental health days and lifetime depression diagnosis among U.S. farming-related occupations versus the general population of people employed in non-farming occupations.

**Methods:**

Data were from 13 states providing industry and occupational data from the Behavioral Risk Factor Surveillance Survey (BRFSS) 2019 survey. Bivariate statistics and logistic regression investigated correlates (sex, race/ethnicity, marital status, education level, and age) associated with poor mental health days in the last 30 days (14 or more days vs. 13 or fewer days) and with a depression diagnosis among farmers versus non-farmers.

**Results:**

The analysis included 55,253 individuals, with 2,773 individuals in farming occupations. In unadjusted models, people in farming occupations were significantly more likely than those in non-farming occupations to be older, White, with lower educational attainment, and a lower prevalence of poor mental health days or depression. In adjusted models, farming and non-farming occupations had no significant difference in the odds of having poor mental health days or a depression diagnosis.

**Conclusions:**

Sociodemographic differences between farming and non-farming occupations echoed previous findings. Being in a farming occupation was not associated with odds of poor mental health days or a depressive disorder diagnosis compared to non-farming occupations, which supports other findings from national datasets. These findings, along with statistics showing a higher rate of suicide among farmers and farmworkers, suggest that additional research is needed about factors related to farm-related occupational wellness and distress.

## Introduction

The U.S. farming population is facing a disproportionately high incidence of suicide,^1–4^ necessitating research on their mental health. Overall, the U.S. suicide rate increased by 35% from 2000 to 2021.^5^ Specifically among farming occupations, the age-adjusted suicide rates from 1986–2014 were 22.3 per 100,000 for farm managers/owners and 21.6 per 100,000 for farmworkers, and rural farmworkers have a 32% higher risk of suicide than all other rural occupations.^4^

Farmer suicide decedents are typically older, White, and male, and they are more likely to be unmarried (single, divorced/separated, or widowed) and have less education than non-farmer decedents.^3,6^ Depression is strongly associated with suicide ideation and attempts,^7^ with approximately half of all suicide decedents having a reported depressive disorder.^8^ Persistent mental distress is often considered a precursor of depression^9,10^ as it diminishes quality of life and well-being^11^ and is associated with suicidality.^10^ According to National Violent Death Reporting System (NVDRS) data, farmer suicide decedents are less likely than the general population of decedents to have had a diagnosed mental health problem or depression before death,^6,12^ although some studies suggest that farmers suffer from depression and anxiety at higher rates than the general population.^13,14^ However, it is unclear whether individuals in farming occupations disclose a higher prevalence of poor mental health days or depression than the general population.

Specific, unique characteristics of individuals in farming occupations may increase their risk for mental distress. For example, their mental distress could be exacerbated by financial difficulties, physical problems or injuries, family issues, unpredictable weather and climate variability that affect yields, equipment and technology challenges, crop and livestock diseases, and geographic isolation.^1,15,16^ Further, it is often difficult for individuals in farming occupations to take days off to recharge and relax, especially if they work with animals or during harvest and planting periods.^16^ Given their distance from neighbors and long hours, community and social events often require extra effort to attend, increasing isolation. Also, working and living in the same place, often with family members, adds stress to individuals in farming occupations.^15^

There are additional contextual stressors in the agricultural environment involving economics and weather. Between 2015 and 2019, farm real estate values declined by 5%, after over a decade of inflation doubling farm values.^17^ By 2017, despite small farms with less than $350,000 in annual revenue making up 89% of U.S. farms, the largest 3% of farms (more than $1,000,000 annual revenue) accounted for 39% of production. In 2019, drought was the leading cause of crop insurance, leading to increased irrigation, especially in the western U.S., where irrigated farms rose from 37% in 1984 to 76% by 2013. In 2019, severe flooding led to widespread crop loss, mostly in the midwestern U.S.^18^

Sociodemographic factors among farmers differ from those of the general population, and these may also affect farmers’ mental health outcomes. Farmers are older than the general population, with a mean age of 58.1 years among producers in 2022, suggesting high aging-related needs.^19^ Further, U.S. producers (95%) are predominantly White,^19^ and farm producers are predominantly male (64%).^19^ In contrast, the U.S. overall population is less White (62%),^20^ has slightly more women than men, and is younger (median age 38.9 years).^21^ With these demographic imbalances, older farmers face uncertainty over the futures of their farms, younger producers may feel increased pressure to succeed,^22^ which leads to enhanced stressors in the existing farmer population.

The Dual-Factor Model of Mental Health posits that occupational stress is a predictor of mental health, including depression and anxiety.^23^ Further, the model views mental health as both encompassing wellbeing factors (emotional and psychological wellness) and the absence of psychopathology (anxiety and depression).^24,25^ Thus, individuals could have poor mental health without a diagnosed disease or symptoms of mental illness. This model, applied to farmers would mean that farmers could face four mental health scenarios: complete health (no psychiatric symptoms and both high well-being), vulnerable (no or few psychiatric symptoms yet poor well-being), symptomatic but content (high levels of psychiatric symptoms yet high well-being), and troubled (poor mental health with low levels of wellbeing).^26^ This model allows for a more nuanced view of mental health within farming, informing tailored interventions, as farmers may experience social and occupation-related stresses (e.g., financial strain, isolation) but not display psychiatric symptoms (e.g., mental illness, suicidal ideation).^6,14^

Given the unique stressors facing the livelihoods of individuals in farming occupations, we sought to further understand the sociodemographic factors and the occupational differences associated with mental distress indicators that may be unique to this industry. Poor mental health includes diagnosed conditions such as depression or anxiety, or it can be described as having emotional difficulties or psychological distress. The Centers for Disease Control and Prevention (CDC) includes assessments of both diagnosed mental health conditions and a self-perceived measure of monthly days of poor mental health (Frequent Mental Distress [FMD]) in its annual survey administered by states, the Behavioral Risk Factor Surveillance System (BRFSS).^27^ A higher level of FMD is positively correlated with depression diagnoses.^27,28^ While the BRFSS is a key national resource for various public health estimates of risks and outcomes, information about people in farming occupations has rarely been studied in these data due to the limited availability of occupational information.

This analysis aims to determine factors associated with the odds of the mental distress indicators of poor mental health days and depression by occupation status of farming versus non-farming work, using industry and occupational data from the BRFSS 2019 survey provided by 13 states. We hypothesized that individuals in farming roles would have more mental distress and higher odds of depression than individuals in non-farming roles. This information and comparison within a major national dataset will inform future research about farming occupations and the factors associated with mental health outcomes.

## Methods

### Design and sample

This secondary analysis utilized data from the BRFSS, an annual, comprehensive health survey administered by each U.S. state. The BRFSS is the most extensive nationwide survey in the U.S., interviewing more than 400,000 adults annually.^29^ In 2019, 25 states collected data about respondents’ industry and occupation, and we were able to gather data from 13 states for the present analysis, including California, Florida, Iowa, Kansas, Louisiana, Mississippi, Montana, New Hampshire, New Mexico, New York, Texas, Vermont, and Wisconsin. The sample represents states from each major Census region of the U.S. These states also represent various levels of agricultural activity and production types, creating a variable sample representing the nation’s farming statistics. The thirteen states included represent 36.3% of all farms in the U.S., with 12.6% of the nation’s 1.9 million farms located in Texas (highest in the nation) and 0.2% of U.S. farms located in New Hampshire (46^th^ highest in the nation).^30^ The 13 states included an average of 53,103 farms in 2022, which is above the national average of 38,010 farms per state, and includes a breadth of production. For example, in 2019, main commodities in Texas were cattle and calves, with $20.2 billion sold; in Iowa, it was corn, hogs, soybeans, and cattle, with $29 billion sold; and in New Hampshire, main products included dairy, apples, and maple syrup with less than $200 million sold.^31^

The response rate for the 13 states in 2019 ranged from a low of 37.3% in New York to a high of 57.7% in Kansas,^32^ and the median response rate was 49.4%. Further details about the methodology of the BRFSS are available from the CDC.^29^ The inclusion criteria for the analysis were individuals who were assessed for occupational data (described in more detail below), and individuals in farming occupations (*n* = 2,773) were compared to individuals who reported non-farming occupations (*n* = 52,480).

### Independent variables

Demographic data utilized in this analysis included age, sex, race, educational level, and marital status. Age is reported as six categories: 18–24, 25–34, 35–44, 45–54, 55–64, and 65 years or older. The BRFSS creates specific variables for use, and one was a combined field for race and ethnicity, which is coded as White, Black, American Indian/Alaska Native, Hispanic, Multiracial, and Asian/Pacific Islander. Marital status was coded as married or partnered, divorced or separated, widowed, or never married. Education level was coded as less than high school, high school graduate, some college, or graduate of a college or technical school.

In states that included the industry and occupation questions, respondents who indicated that they were either self-employed, working for wages, or out of work for less than 1 year, were asked: “What kind of work do you do?” and “What kind of business or industry do you work in?”. The BRFSS interviewer recorded a summary of each respondent’s answers. From those brief, open-ended answers, farming occupations were identified. These answers were recorded using detailed farming National Health Interview Survey (NHIS) codes for occupation and industry, North American Industry Classification System (NAICS) codes, and self-described industry and occupation data. Occupational codes were provided by 10 states: Iowa, Louisiana, Mississippi, Montana, New Hampshire, New Mexico, New York, Texas, Vermont, and Wisconsin. Only the brief qualitative text that the interviewer recorded based on the respondents’ answers to occupational questions was used for states that did not provide occupational codes: California, Florida, and Kansas. Although the self-described occupational answers were self-explanatory, two authors reviewed the brief, self-described occupational text to ensure consistency in coding farming occupations. In this study, farming occupations encompassed all individuals in agriculture roles, including farmworkers, support roles, and operation owners.

### Dependent variables

In the BRFSS survey, the following questions^33^ assess mental health and depression: 1) “Now thinking about your mental health, which includes stress, depression, and problems with emotions, for how many days during the past 30 days was your mental health not good?” asking for a number between zero and 30, and 2) “(Ever told) (you had) a depressive disorder (including depression, major depression, dysthymia, or minor depression)?” The number of poor mental health days per month was entered as a numerical value in BRFSS surveys, but it was not normally distributed. Therefore, it was dichotomized to be 14 or more days of self-reported poor mental health days in the previous 30 days (FMD-14), as this outcome is commonly used by the CDC HRQOL-14 and in multiple population health surveys and surveillance systems.^34^ FMD-14 also represents the burden of mental illness in multiple populations.^28^ Lastly, the depression question was coded into a dichotomy of lifetime depressive disorder diagnosis (yes/no).

### Data analysis

Descriptive statistics were used to identify missing values and characterize the total sample, including individuals identifying as farming and non-farming occupational groups. Missing values were categorized as “unknown” and retained in the analysis. These groups were compared using Pearson’s chi-squared tests. Due to the survey weights, chi-square test results are reported with a second-order Rao and Scott correction, which generates a Wald-design-based F-statistic with degrees of freedom that can contain decimals (i.e., non-integers).^35^

To determine the prevalence of poor mental health days, logistic regression was used to examine factors related to the dichotomized FMD-14 outcome. Factors related to a depressive disorder diagnosis were also analyzed by logistic regression, and all models were adjusted for the sociodemographic covariates and occupation type. Estimates are reported as adjusted odds ratios along with 95% confidence intervals. All data cleaning, coding, and analyses were performed using Stata/MP version 18 software. All analyses accounted for the complex sampling design of the BRFSS, utilizing the national weights to address noncoverage and nonresponse. Weights for this analysis employed primary sampling unit and strata, and we employed Taylor series linearization for variance estimation. More detailed information about the BRFSS weights is available from the CDC.^36^ Statistical significance was assessed at *p* < .05 for all tests. Because BRFSS data are deidentified and publicly available through the CDC, the University of Southern California institutional review board considered this project not human subjects research.

## Results

The percentages of farming occupations for the 13 states included closely matched those of other estimates.^30^ For instance, the Iowa 2019 BRFSS sample consisted of 4.4% (*n* = 434) individuals in farming occupations, while 2025 estimates show that 4% of the state’s labor force is employed in farm occupations.^37^

In unadjusted analyses, individuals in farming occupations had a significantly higher prevalence of identifying as White or Hispanic (χ^2^ (4.16) = 2.54, *p* = .04, Cramér’s V = 0.03), 45 years or older (χ^2^ (4.76) = 3.19, Cramér’s V = 0.03), and have less than a high school education than non-farming occupations (χ^2^ (3.48, *N* = 115,249) = 5.66, *p* < .001, Cramér’s V = 0.04) (Table 1). Compared to non-farming occupations, individuals in farming occupations had a higher (but not statistically significantly higher) prevalence of males (*p* = .08). There were no statistically significant differences between individuals in farming vs. non-farming occupations regarding the prevalence of being married, partnered, or divorced (*p* = .17). For FMD-14, 9.9% of individuals in farming occupations reported frequent mental distress days, compared to 11.8% among non-farming occupations, which was not statistically significantly different (*p* = .13). Similarly, there was no statistically significant difference between farming (13.0%) and non-farming (14.6%) occupations for a depressive disorder diagnosis (*p* = .26). Cramér’s V values ranged from 0.02 to 0.04, indicating weak associations.^38^

Each of the full models for the two separate outcomes was statistically significant (*p* < .001), showing that the model could distinguish between having poor mental health days (FMD-14) or not, or having a depressive diagnosis or not. Table 2 shows the significant predictors in the models.

There was no statistical difference between farmers and non-farmers in the odds of FMD-14. Significant factors for the model predicting FMD-14 included age, sex, race, marital status, and education level. Compared to the 18–24 year group, all older age groups had lower odds of FMD-14, with ages 25–34 years (aOR = 0.71; 95%CI-0.58–0.89), ages 35–44 years (aOR = 0.56; 95%CI = 0.44–0.71), ages 45–54 years (aOR = 0.42; 95%CI = 0.32–0.54), ages 55–64 years (aOR = 0.43; 95%CI = 0.32–0.58), and ages 65 or older (aOR = 0.24; 95%CI = 0.16–−0.36). Females had higher odds of FMD-14 than males (aOR = 1.54; 95%CI = 1.35–1.75). Black (aOR = 1.32; 95%CI = 0.54–0.99), Asian (aOR = 0.54; 95%CI = 0.35–0.82), and Hispanic (aOR = 0.75 95%CI = 0.62–0.91) individuals were significantly less likely to have FMD-14 than White individuals. Compared to married individuals, divorced/separated (aOR = 2.07; 95%CI = 1.73–−2.48), widowed (aOR = 1.53; 95%CI = 1.13–2.08), and never married (aOR = 1.64; 95%CI = 1.39–−1.94) individuals had significantly higher odds of FMD-14. Compared to individuals with less than a high school education, individuals with some college or technical school (aOR = 0.77; 95%CI = 0.60–0.99) or who had graduated from college or technical school (aOR = 0.54; 95%CI = 0.42–0.70) had significantly lower odds of FMD-14. The Hosmer-Lemeshow test was significant and the pseudo-R^2^ (McFadden’s) statistic was 0.05. The Variance Inflation Factor (VIF) was 1.97, indicating little multicollinearity among predictors, considered acceptable.

For the model predicting odds of depressive disorder diagnosis, there was no difference between farming or non-farming occupations. Significant factors included age, sex, race, and marital status. Compared to individuals ages 18–24 years, individuals ages 45–54 years (aOR = 0.71; 95%CI = 0.57–0.88), 55–64 years (aOR = 0.65; 95%CI = 0.51–0.82), and 65 years and older (aOR = 0.47; 95%CI = 0.37–0.61) had lower odds of having a depression diagnosis. Females had higher odds of depression than males (aOR = 2.15; 95% CI = 1.93–2.39). Black (aOR = 0.39; 95%CI = 0.32-–0.47), American Indian or Alaska Native (aOR = 0.68; 95%CI = 0.46–1.00), Asian (aOR = 0.28; 95% CI = 0.18–0.44), and Hispanic (aOR = 0.47; 95% CI-0.40–0.55) individuals were significantly less likely to have a depression diagnosis than White individuals. Divorced/separated (aOR = 1.95; 95% CI-1.69–2.25), widowed (aOR = 1.30; 95%CI = 1.02–1.65), or never-married individuals (aOR = 1.74; 95%CI = 1.52–2.01) had significantly higher odds of depression than married individuals. The odds of a depression diagnosis did not significantly differ by level of education. An unweighted model was run to conduct additional model diagnostic tests. The Hosmer-Lemeshow test was significant, and the pseudo-R^2^ (McFadden’s) statistic was 0.05. The Variance Inflation Factor (VIF) was 1.96, indicating little multicollinearity among predictors, considered acceptable.

## Discussion

This study analyzed factors related to having poor mental outcomes (i.e., FMD-14 and lifetime depressive disorder diagnosis), comparing individuals in farming to those in non-farming occupations assessed by the BRFSS. The findings revealed that being in a farming occupation was not significantly associated with poor mental health indicators. The present findings stand somewhat in contrast to other research indicating a disproportionately higher suicide rate^2,3,6^ for people in farming occupations, which would suggest that mental health indicators should be poorer among this group compared to individuals in non-farming occupations. In the following discussion, we more deeply consider these findings in light of extant research.

The present findings echo some previous studies comparing the mental health outcomes of farmers to those of the general population, with a review of farmer mental health measures showing that farmer mental health outcomes are better or equivalent to those of non-farmers.^39^ Still, other studies have suggested that farmers have higher levels of depression and anxiety than the rest of the population,^39,40^ such as the study by Rudolphi et al. They used a small, self-selected group of young farmers in the U.S. Midwest (*n* = 170) and found that the farmers’ PHQ-9 and GAD-7 scores were higher than those for U.S. population norms. A systematic review that included 28 articles comparing farmers’ mental health to non-farmers found that 71% of these studies indicated worse mental health in farmers.^15^ Qualitative research about farmers’ mental health also indicates that they have high levels of stress.^41^ Studies of rural mental health suggest numerous structural deficits in rural areas (compared to urban areas) that could be associated with poor mental health, including lack of access to healthcare, greater loneliness, and greater isolation.^42,43^ Yet some studies’ findings indicated no robust urban-rural differences in self-reported mental health metrics.^44^ For example, using data from the Midlife in the U.S. (MIDUS) and the Health and Retirement Study (HRS), Atherton et al. found that initial, unadjusted urban-rural differences in self-reported psychological well-being were no longer statistically significant after adjusting for other sociodemographic covariates.^45^ The dissonance across studies requires further research, such as determining measurement differences or harmonizing different mental health measures for aggregate analyses.

Depression diagnoses are common antecedents to suicide,^7^ with upwards of 90% of suicide deaths having co-occurring mental illness (mostly depression).^8^ Miller and Rudolphi found that farmer suicide decedents had no difference in the prevalence of mental illness or depressive issues compared to non-farmer suicide decedents.^6^ The present results appear to echo those equivocal findings about mental health and depression among living respondents, showing no significant difference between farming and non-farming populations. A Danish study suggested that although farmers may have a higher prevalence of depression and suicide, there is a weaker association among farmers between depression and increased mortality than in other occupational groups.^46^ These findings suggest that perhaps people in farming occupations, despite having many stressors, may also have many unique strengths and resilience that may buffer these stressors. For example, research has identified how social support impacts the well-being of farmers, identifying that non-family support buffers social stress,^47,48^ and relationships with farming peers are vital to build resiliency.^49,50^ Additional studies revealed that religious practices can improve farmer psychological health^16,51,52^ and that a connection to the land increases resilience.^53,54^

Despite the lower although not significantly different prevalence of typical poor mental health indicators in the outcome data for individuals in farming-related occupations, other research indicates they have a higher-than-average suicide rate.^2,6^ Other studies note this incongruence between mental health outcomes and suicide rates among farmers,^39^ given that mental and psychiatric diagnoses, including depression and anxiety, are risk factors for suicide.^55^ This seeming dissonance invites several potential, testable explanations for future research. One potential explanation may be that individuals in farming reveal few of the typical warning signs that precede suicide, which is consistent with other studies revealing scant endorsements of suicidal ideation among farmer decedents.^14,39^ Altogether, these findings, along with farmer-specific stressors, suggest that individuals in farming occupations may progress differently toward suicide than other populations (e.g., having fewer mental health warning signs). For instance, distress may be an acute response to stressors combined with contextual factors, rather than as the result of psychiatric issues.^39^

The lack of statistically significantly different prevalences of depression and frequent mental distress among individuals in farming occupations may also be partially explained by farmers’ access to and utilization of mental healthcare and their culture and beliefs, which include a strong sense of independence and pride in their work, as many farmers view themselves as stewards of the land and as sustainers of the nation with food.^41,56^ Studies demonstrate that even if farmers have access to mental healthcare, they may underutilize it, especially given perceptions of it being inaccessible, long distances to travel for care, and the pervasive stigma toward receiving mental healthcare,^57,58^ resulting in fewer mental health diagnoses among farming populations. Further nuancing questions around farmers’ disproportionately high suicide rate, individuals in farming occupations express higher levels of religiosity, which is protective against suicide, than individuals in other occupations.^59^ Also, farmers are less likely than the general population to endorse suicide as acceptable in certain circumstances (bankruptcy, dishonoring family, etc.).^60^ Given these seemingly paradoxical findings and underreporting of typical predisposing factors to suicide (e.g., mental distress), additional exploration into self-reported mental health measures among farmers is needed, perhaps with greater attention to how farmers, themselves, define and refer to mental distress (i.e., idioms of distress).^61^

Findings around sociodemographic factors in this sample were consistent with those from other studies. For example, the present results found that females had a higher prevalence and adjusted odds ratio of frequent mental distress and depression diagnoses than males. This was consistent with other studies examining FMD and mental disorders in general and chronically ill populations.^62,63^ Most (80%) suicides in the U.S. are among males,^5^ however, suggesting that FMD and depression may be underreported among men.^2,6^ In the adjusted models, unmarried or unpartnered individuals were significantly more likely to have FMD or depressive disorders, which echoes other studies’ findings that unmarried/unpartnered individuals are more likely to have mental distress.^62,63^ Other studies have demonstrated that farmer suicide decedents are less likely than non-farmer decedents to be married or partnered,^2,6^ and overall U.S. suicide statistics^64^ indicate that marriage or domestic partnership is protective against suicide, suggesting that individuals in farming occupations who are not married or partnered require extra social support.

To our knowledge, this is the only analysis incorporating occupational data from the BRFSS to examine differences between farming occupations and the general population. Despite the strengths of this analysis having a large, probability-based sample of farming occupations from geographically diverse regions of the U.S., there are limitations. This is an incomplete view of the U.S., as several states with significant numbers of farmers were not included due to a lack of data availability, which limits generalizability. The standard cut-off of 14 days of poor mental health was used as an outcome measure, and this dichotomous approach may have limited variance.

Further, as a cross-sectional study, causal inferences cannot be made about BRFSS data, and despite finding some significant associations, certain statistical tests indicate that these should be interpreted with caution. For the weighted unadjusted analyses, the effect sizes (Cramér’s V) were low, suggesting minor associations. For the unweighted logistic regressions, the McFadden’s pseudo R^2^ was low, and the Hosmer-Lemeshow statistic was significant, indicating poor model fit.^65^ As mental health outcomes are multifactorial and notoriously complex to predict, these statistics suggest that further research into their predictors is needed.

In addition to differences in farm outputs, practices, and commodities, there is variation in mental health resources for farmers by state, further affecting the interpretation of this study’s results. Individual states from the dataset also have variable farmer-specific mental health programs and services. Currently, all the states included in the sample had at least a webpage with resources aimed at farmer mental health and suicide prevention, either a dedicated, extension-based, or a farm bureau-based, or agriculture department program, and several with farmer-specific telephone hotlines, including those in Vermont, Wisconsin, and Texas.^66^

Occupational data were self-described by the interviewees, which may have limited quality due to recall bias, response bias, and social desirability. BRFSS respondents are limited to noninstitutionalized individuals, which may introduce sampling bias and limit generalizability. Farmworkers were not differentiated from farm operators, who may have distinct stressors. Also, many individuals who farm also have off-farm occupations,^13^ which they may have used to self-describe their industry and occupation instead of listing farming, introducing misclassification bias and undercounting individuals in farming occupations. The occupational data provided by the 13 states did not use consistent fields, prohibiting a closer analysis comparing farm roles (e.g., farmer, farmworker, family members, and support roles) or different farm types (e.g., arable vs. livestock). Further, especially for states providing open-ended occupational data or limited industry codes, it was impossible to differentiate farm operators from farmworkers (e.g., many self-described as “FARM”).

## Conclusion

This study fills an important gap in the literature surrounding the mental health of individuals in farming occupations by utilizing population-level mental health surveillance indicators. Future research could include larger samples of occupational data from national datasets, and specific studies that compare mental health outcomes and other correlates between non-farming and farming occupations, ideally differentiating between farm operators and workers. While the results do not indicate that individuals in farming demonstrate the typical indicators of poor mental health, this study can inform the public health approach to health promotion by determining further research and interventions to identify relevant issues and ultimately address the mental health of individuals in farming occupations with targeted interventions.

## Figures and Tables

**Table 1.** Prevalence and unadjusted differences in socio-demographics, poor mental health days, and lifetime depressive disorder diagnosis by farming versus non-farming occupations, BRFSS 2019, 13 states.

*Socio-demographics*	Full sample		Individuals in farming occupations		Individuals not in farming occupations		
	*N* = 55,253		*n* = 2,773		*n* = 52,480		
	n	(%)	n	(%)	n	(%)	*p*
**Gender**							
Women	28,921	(49.5)	1,110	(45.8)	27,811	(49.6)	0.08
Men	26,332	(50.5)	1,663	(54.2)	24,669	(50.4)	
**Race**							
White	39,874	(50.6)	2,102	(51.6)	37,772	(50.6)	0.04
Black	3,807	(11.1)	149	(11.4)	3,658	(11.1)	
Amer.Indian/Alaska Native	971	(0.01)	34	(0.6)	937	(0.8)	
Asian	1,019	(5.7)	20	(2.6)	999	(5.9)	
Multiracial	929	(0.01)	32	(0.7)	897	(1.0)	
Hispanic	7,215	(28.5)	374	(31.6)	6,841	(28.4)	
Other/Unknown	1,438	(2.3)	62	(1.5)	1,376	(2.4)	
**Age group**							
18–24 years	3,666	(11.2)	157	(9.0)	3,509	(11.3)	< 0.01
25–34 years	9,088	(23.0)	366	(19.7)	8,722	(23.2)	
35–44 years	10,244	(22.2)	435	(20.5)	9,809	(22.3)	
45–54 years	11,411	(20.3)	517	(21.2)	10,894	(20.2)	
55–64 years	12,731	(16.1)	703	(20.3)	12,028	(16.0)	
65+ years	8,113	(7.1)	595	(9.3)	7,518	(7.0)	
**Marital status**							
Married/partnered	33,557	(59.8)	1,826	(60.5)	31,731	(59.8)	0.17
Divorced/separated	8,215	(12.3)	377	(14.3)	7,838	(12.2)	
Widowed	2,678	(2.6)	125	(2.7)	2,553	(2.6)	
Never married	10,404	(24.5)	434	(22.3)	9,970	(24.6)	
Unknown	395	(0.8)	11	(0.3)	384	(0.9)	
**Education level (highest)**							
< High school	3,725	(12.9)	232	(18.9)	3,493	(12.7)	< .001
High school graduate	14,141	(26.7)	723	(24.1)	13,418	(26.8)	
Some college/tech school	15,638	(30.1)	783	(30.0)	14,585	(30.2)	
Graduate coll./tech school	21,809	(29.7)	1,026	(26.8)	20,783	(29.8)	
Unknown	210	(0.5)	9	(0.2)	201	(0.5)	
***Mental Health Indicators***							
FMD-14							
< 14 days/past 30 days	48,302	(88.3)	2,515	(90.1)	45,787	(88.2)	0.13
≥14 days/past 30 days	5,965	(11.7)	218	(9.9)	5,747	(11.8)	
**Lifetime depressive disorder diagnosis**							
Yes	8,902	(14.6)	315	(13.0)	8,587	(14.6)	0.26
No	46,115	(85.0)	2,448	(86.7)	43,667	(84.9)	
Unknown	236	(0.5)	10	(0.3)	226	(0.5)	

Notes: Pearson’s Chi-Squared test; FMD = frequent mental distress.

**Table 2.** Adjusted odds of having 14 or more poor mental health days/past 30 days or a life lifetime depressive disorder diagnosis, by farming versus non-farming occupation and socio-demographics, BRFSS 2019, 13 states.

	FMD-14 (*n* = 54,263)		Lifetime depressive disorder diagnosis (*n* = 55,013)	
	aOR	(95%CI)	aOR	(95%CI)
Non-farming occupation	Ref		Ref	
Farming occupation	0.85	(0.64–1.12)	0.90	(0.72–1.13)
**Age group**				
18–24 years	Ref		Ref	
25–34 years	0.71	(0.58–0.89)*	0.91	(0.75–1.11)
35–44 years	0.56	(0.44–0.71)*	0.87	(0.70–1.07)
45–54 years	0.42	(0.32–0.54)*	0.71	(0.57–0.88)*
55–64 years	0.43	(0.32–0.58)*	0.65	(0.51–0.82)*
65+ years	0.24	(0.16–0.36)*	0.47	(0.37–0.61)*
**Sex**				
Male	Ref		Ref	
Female	1.54	(1.35–1.75)*	2.15	(1.93–2.39)*
**Race**				
White	Ref		Ref	
Black	0.81	(0.65–0.99)*	0.39	(0.32–0.47)*
Amer. Ind./AK Native	1.32	(0.73–2.37)	0.68	(0.46–1.00)*
Asian	0.54	(0.35–0.82)*	0.28	(0.18–0.44)*
Multiracial	1.14	(0.86–1.51)	1.06	(0.80–1.40)
Hispanic	0.75	(0.62–0.91)*	0.47	(0.40–0.55)*
Other/Unknown	1.05	(0.73–1.51)	0.72	(0.50–1.02)
**Marital status**				
Married/partnered	Ref		Ref	
Divorced/separated	2.07	(1.73–2.48)*	1.95	(1.69–2.25)*
Widowed	1.53	(1.13–2.08)*	1.30	(1.02–1.65)*
Never married	1.64	(1.39–1.94)*	1.74	(1.52–2.01)*
Unknown	2.18	(1.17–4.04)*	1.66	(0.85–3.24)
**Education level**				
< High school	Ref		Ref	
High school graduate	0.78	(0.61–1.01)	0.88	(0.70–1.10)
Some college/tech. school	0.77	(0.60–0.99)*	1.06	(0.85–1.33)
Graduate college/tech. school	0.54	(0.42–0.70)*	0.90	(0.72–1.12)

Note: All models adjusted for occupation, age, sex, race/ethnicity, marital status, and education.

* = p < .05.

FMD = frequent mental distress.
